# Addition of lactoferrin increases efficacy of three Kayviruses and limits the inflammatory response in pulmonary epithelial cells

**DOI:** 10.1007/s00253-025-13695-9

**Published:** 2026-01-10

**Authors:** Katarzyna Kosznik-Kwaśnicka, Grzegorz Stasiłojć, Milena Grzenkowicz, Małgorzata Stasiłojć, Agnieszka Necel, Lidia Piechowicz

**Affiliations:** 1 Department of Medical Microbiology, Faculty of Medicine, Medical University of Gdansk, Debowa 25, 80-204, Gdansk, Poland; 2 Department of Cell Biology and Immunology, Intercollegiate Faculty of Biotechnology of University of Gdansk and Medical University of Gdansk, Debinki 1, 80-211, Gdansk, Poland; 3 Department of Microbiology, Faculty of Biology, University of Gdansk, Wita Stwosza 59, 80-308 Gdansk, Poland

**Keywords:** Lactoferrin, Bacteriophage, *Staphylococcus aureus*, Phage therapy, A549

## Abstract

**Abstract:**

*Staphylococcus aureus* is a major cause of hospital-acquired pneumonia, with methicillin-resistant strains contributing significantly to prolonged illness and mortality. Methicillin-resistant strains can be responsible for up to 75% of infections in certain countries. Therefore, the problem is described as severe, and the search for alternative methods of treatment of such infections is currently one of the priorities in healthcare. Bacteriophages, although historically underutilized, are re-gaining interest for their potential in treating bacterial infections. However, they do have their limitations such as specific ranges of activity and resistance development. Combining phages with antimicrobial agents such as lactoferrin—a natural protein with antimicrobial and anti-biofilm properties—may improve treatment outcomes. In this study, we evaluated the efficacy of three Kayviruses paired with lactoferrin against MRSA in infected pulmonary epithelial cell cultures. The combination significantly reduced bacterial viability, protected human cells from cytotoxic effects of bacterial infection, and decreased inflammasome activation. These findings suggest that phage-lactoferrin combinations may offer a promising, safer alternative for managing MRSA-related pneumonia and reducing dependence on traditional antibiotics.

**Key points:**

•*Phage lactoferrin mixture had no influence on A549 cells*

•*Lactoferrin increased phage efficacy and reduced influence of bacteria on cells*

•*Phage + Lf mixture limited inflammatory response similarly to phages and Lf alone*

## Introduction

Pneumonia caused by *Staphylococcus aureus* accounts for 20–50% cases of hospital acquired pneumonia (HAP) (Calle et al. 2016; Alwakeel et al. 2025). Infections caused by this pathogen can be particularly severe if they are a consequence of primary viral infections such as influenza or SARS-CoV-2 (Mulcahy and McLoughlin 2016; Piechowicz et al. 2024). The emergence of antibiotic resistance among hospital strains adds to severity of infections and is responsible for prolonged hospitalization and increased mortality (Thompson et al. 1982). Perhaps, the most important resistance mechanism detected in *S. aureus* is the resistance to methicillin. Those strains are identified as MRSA (methicillin-resistant *S. aureus*), and their pooled global prevalence is estimated at ~ 14.69% (Hasanpour et al. 2023). However, rates vary from country to country, ranging from a small percent in Scandinavian countries to 50–75% in the U.S. and Asian countries (Cheung et al. 2021; Petersen et al. 2021). In addition to resistance to antibiotics *S. aureus* also has the ability to adhere to human tissue, including respiratory tract epithelial cells, adding to its virulence (Piechowicz et al. 2024; Siboo et al. 2001; Mongodin et al. 2002; Schröder et al. 2006). Adhesion may lead to biofilm formation, which may lead to more complications in treatment, as bacteria in the biofilm are more resistant to eradication (Lamret et al. 2020; Theis et al. 2023). Furthermore, cells that detach from mature biofilm spread throughout organism and may be responsible for additional complications such as sepsis (Minasyan 2019). Therefore, in order to reduce dependence on antibiotics, other alternatives such as enzymes, bacteriophages, metals, or peptides are being explored (Kumar et al. 2021; Ruiz-Pérez et al. 2024).

Bacteriophages, viruses that infect bacteria, have been developed to combat bacterial infections over 100 years ago (Summers 2012). However, it had never become the primary treatment method and remained largely unknown, except in few countries such as Georgia or Poland (Parfitt 2005; Górski et al. 2020). As there has been a significant increase in research on the therapeutic potential of phages in the last 20 years, the number of case studies reporting successful phage treatment of patients even with severe infections such as pneumonia or sepsis has been growing (Lebeaux et al. 2021; Żaczek et al. 2022). Nevertheless, phage therapy is not without its limitations, such as a narrow spectrum of activity, preference for metabolically active bacteria, or development of resistance mechanisms to phages (Górski et al. 2020; Loc-Carrillo and Abedon 2011; Lin et al. 1857). All this may hinder the efficiency of phages and influence the treatment outcome in a negative way. In order to increase phage efficacy and limit undesirable interactions, one of the proposed recommendations is to pair bacteriophages with other antimicrobials (Loganathan et al. 2024). Since the global consensus is to reduce the use of antibiotics, other compounds are being researched (Ghosh et al. 2016; Walsh et al. 2021). Lactoferrin (Lf), a protein that is a part of mammal innate immunity, has documented antimicrobial, and anti-biofilm activity and may be a good candidate for a phage-antimicrobial cocktail (Farnaud and Evans 2003; Zimecki et al. 2008). In our previous works, we have observed that phage-lactoferrin pairing showed antibiofilm activity against multi-drug resistant clinical strains of *S. aureus* (Kosznik-Kwaśnicka et al. 2022). Furthermore, we have also observed that lactoferrin increased the phage efficacy of plating and burst size, which may increase the efficacy of potential future treatment (Grzenkowicz et al. 2025a). Therefore, we have decided to analyze the efficacy of three Kayviruses paired with lactoferrin in MRSA-infected cultures of pulmonary epithelial cells. We have analyzed the ability of the mixture to reduce the number of viable bacterial cells, as well as the influence of the treatment on the metabolic condition of human cells and activation of the inflammatory response. We have observed that the use of phage-lactoferrin cocktail was able to reduce the number of bacterial cells in the culture while inhibiting the cytotoxic effect of *S. aureus* on human cells. Furthermore, the use of the cocktail reduced inflammasome activation to lower levels than the use of its components separately. Therefore, it could be assumed that supplementation of phage lysates with lactoferrin may increase the efficacy of the treatment, without negative influence on the patient’s health. However, more studies on the nature of this phenomenon are needed to fully evaluate its potential.

## Materials and methods

### Human cell line and culture conditions

Human carcinoma lung epithelial cell line A549 (ATCC, CCL-185) (ATCC, Manassas, VA, USA) were cultured in 75-cm^2^ Culture Flask (Corning, New York, NY, USA) in a F-12 K culture medium (ATCC, Manassas, VA, USA) supplemented with 10% fetal bovine serum (FBS; ATCC, Manassas, VA, USA), at 37 °C in a humidified atmosphere of 95% air and 5% CO_2_ in the HeraCell 150 incubator (Heraeus, Hanau, Germany) in accordance with the supplier’s guidelines.

### Bacteriophages

Bacteriophages vB_SauM-A, vB_SauM-C, and vB_SauM-D were isolated from wastewater and were previously characterized (Kosznik-Kwaśnicka et al. 2022; Łubowska et al. 2019). For phage purification, polyethylene glycol (PEG) 8000 (BioShop, Burlington, ON, Canada) was added to phage lysate (final concentration 10% w/v) and stirred overnight at 4 °C. The precipitate was collected by centrifugation at 10,000 × g for 20 min at 4 °C and suspended in F12-K cell medium. PEG 8000 was removed by adding an equal volume of chloroform and centrifugation at 3000 × g for 15 min. The procedure was repeated until no PEG 8000 residue was observed after centrifugation. After the PEG 8000 was removed, FBS was added to the medium to a final concentration of 10%. Purified lysates were stored at 4 °C.

### Lactoferrin

Lactoferrin from bovine milk (Sigma-Aldrich, St. Louis, MO, USA) was dissolved in F-12 K medium and filtered through a 0.22 µm cellulose acetate filter (Merc, Darmstadt, Germany) to form a stock solution of 10 mg/mL. The stock solution was then stored at 4 °C for up to a week.

### Bacterial strains

Three multi-drug-resistant *Staphylococcus aureus* clinical isolates were chosen from the collection of the Department of Medical Microbiology, the Medical University of Gdańsk. The strains were previously described and characterized and will be referred to in this publication as MRSA strains no. 70, 110, and 203 (Łubowska et al. 2019).

### Assessment of phage and lactoferrin activity in infected cell culture

*S. aureus* strains were grown in Nutrient Broth (BioShop, Burlington, ON, Canada) as follows: an overnight culture of the strain was added to fresh medium at a 1:50 ratio and then incubated with shaking at 37 °C, 150 rpm until the optical density of OD_600_ = 0.1. One milliliter of culture was then centrifuged for 5 min at 4 °C and 1000 × g. The supernatant was discarded, and the pellet was washed with 0.9% NaCl. Bacteria were then suspended in 1 mL of F12-K medium and used immediately in cell culture.

A549 cells were plated into 96-well tissue culture plates (Nest Scientific Biotechnology, Wuxi, China) at a density of 10^4^ per well and allowed to attach for 24 h in F12-K supplemented with 10% FBS. The medium was then removed, and the cells were infected with 10^4^ CFU/mL (Colony-Forming Unit/mL) of chosen *S. aureus* strain. Two hours post-infection, bacteriophages (10^9^ plaque-forming unit or PFU/mL), lactoferrin (1 mg/mL), or linezolid (2 mg/mL) was added. A set of three wells per replicate was left untreated as a control. After 3 and 6 h of incubation, the medium was removed and serial dilutions were made in 0.9% NaCl. Fifty microliters of each dilution was spread on Nutrient Agar plates. The samples were incubated overnight at 37 °C and then scanned for colonies for CFU/mL count.

### Influence of bacteriophages and lactoferrin on cell viability using neutral red staining

The assay was performed in accordance with the protocols previously published with some modifications (Repetto et al. 2008). The cells were treated as described in Sect. “Assessment of phage and lactoferrin activity in infected cell culture.” Additionally, a set of cells was treated with 10% DMSO (Sigma-Aldrich, St. Louis, MO, USA) as a positive control. Following incubation, the supernatant was discarded and replaced with 100 μL of F-12 K supplemented with a 0.33% neutral red solution (Sigma-Aldrich) at a 1:40 ratio. After 2 h of incubation at 37 °C, the medium was removed, and the cells were washed with 100 μL of the phosphate-buffered saline (PBS) per well. Subsequently, the cells were treated with 150 μL of a de-staining solution (50% ethanol, 49% distilled H_2_O, and 1% acetic acid [Alchem, Torun, Poland]) and incubated with shaking at 37 °C for 10 min. Absorbance was measured at 540 nm (Synergy H1, BioTek Instruments, Winooski, VT, USA).

### Influence of bacteriophages and lactoferrin on integrity of cell membranes

A549 cells were plated and treated with bacteriophages, lactoferrin, or linezolid as described in Sect. “Assessment of phage and lactoferrin activity in infected cell culture.” The LDH (lactate dehydrogenase) release assay was then performed using CytoTox 96® (Promega, Madison, WI, USA) in accordance with the manufacturer’s protocol.

### Influence of bacteriophages and lactoferrin on inflammasome activation

A549 cells were plated and treated with bacteriophages, lactoferrin, or linezolid as described in Sect. “Assessment of phage and lactoferrin activity in infected cell culture.” To measure the level of inflammasome activation, Caspase-Glo® 1 Inflammasome Assay (Promega, Madison, WI, USA) was used in accordance with the manufacturer’s protocol.

### Phage transcytosis

The experiment was designed based on previously published protocols with minor modifications (Dąbrowska et al. 2009; Nguyen et al. 2017). The upper surface of 0.4-µm Translucent High Density PET Membrane transwell inserts (Falcon®, Falcon Biotech Pvt Ltd, Punjab, India) was coated with 10 µl of Matrigel® Membrane Matrix (Corning, New York, USA), diluted 1:4 in F-12 K medium, and allowed to dry overnight at 37 °C in a covered plate. A549 cells suspended in F12-K supplemented with 10% FBS were then seeded on both: the bottom of the well (2 × 10^4^ per well) and the Matrigel®-coated membrane (1 × 10^4^) and incubated for 24 h to allow attachment. Following incubation, the culture medium was replaced and phages were added to the upper chamber at a final concentration of 10^9^ PFU/mL. The migration was assessed after 24 h. To this end, the medium from the upper chamber was discarded, and the cells on the upper side of the membrane were removed with a cotton swab. The medium from the lower chamber was collected and serially diluted in TM buffer. Cells from the lower chamber were also collected using cotton swabs. The swabs were then placed in TM buffer and incubated for 30 min at room temperature; then the buffer was serially diluted. The dilutions were then titrated using a double-layered plate technique in order to calculate the PFU/mL (Grzenkowicz et al. 2025a).

## Results

### Influence of phages vB_SauM-A, vB_SauM-C, vB_SauM-D, and lactoferrin on cell viability

**Fig. 1:**
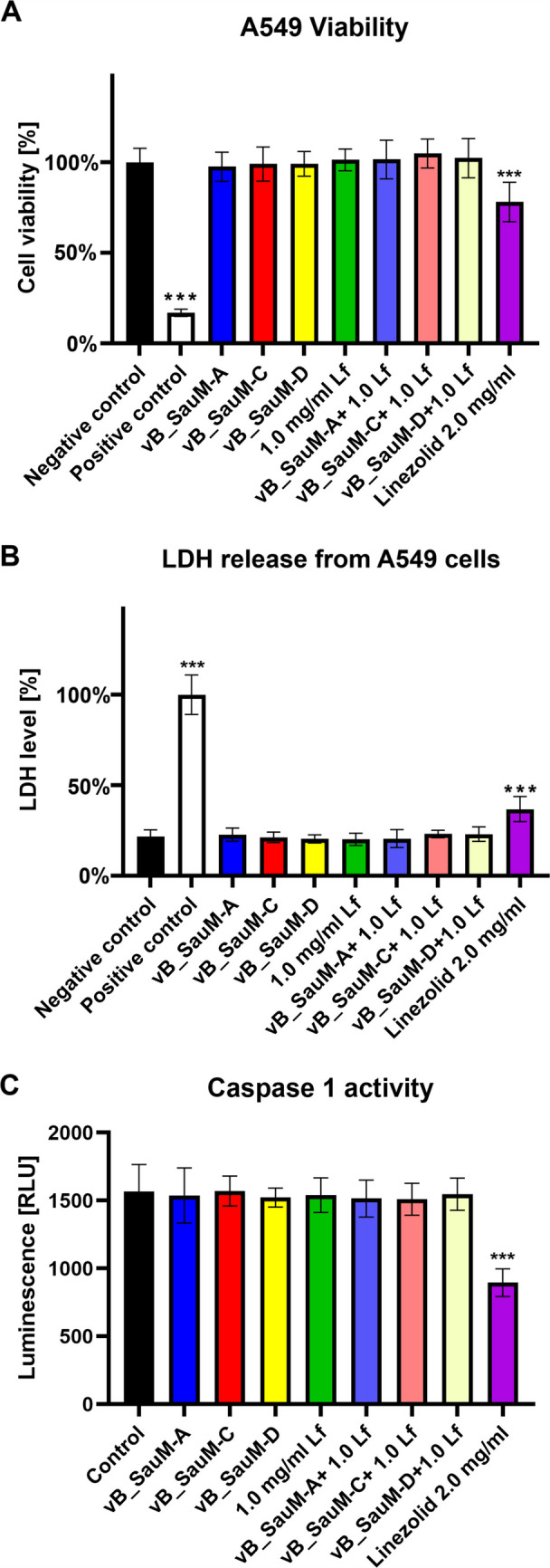
Cell viability (**A**), LDH release (**B**), and caspase-1 activity (**C**) of the A549 cells treated with phages vB_SauM-A, vB_SauM-C, vB_SauM-D, 1.0 mg/mL lactoferrin, 2.0 mg/mL linezolid, and bacteriophages supplemented with 1 mg/mL lactoferrin in comparison to the untreated negative control and positive control. Arithmetic mean of triplicates with error bars representing SD. Statistical analysis was performed using one-way ANOVA, with ***p < 0.001

Based on previously published research, the working concentrations were established at 1 mg/mL for lactoferrin and 1 × 10^9^ PFU/mL for phages (Kosznik-Kwaśnicka et al. 2022; Grzenkowicz et al. 2025a). Additionally, we have decided to compare the influence of phages and Lf with linezolid, an antibiotic that is used in treatment of MRSA-caused respiratory tract infections. We have observed that bacteriophages, Lf, or combination of both did not influence the viability (measured by neutral red) or integrity of cell membranes (measured by LDH release) (Fig. 1A, B). None of the tested combinations also seemed to increase caspase-1 levels that would suggest inflammasome activation (Fig. 1C). On the contrary, the use of linezolid resulted in ~ 20% decrease in both, cell viability as well as inflammasome activation (Fig. 1).

### Assessment of phage and lactoferrin activity in infected cell culture

**Fig. 2:**
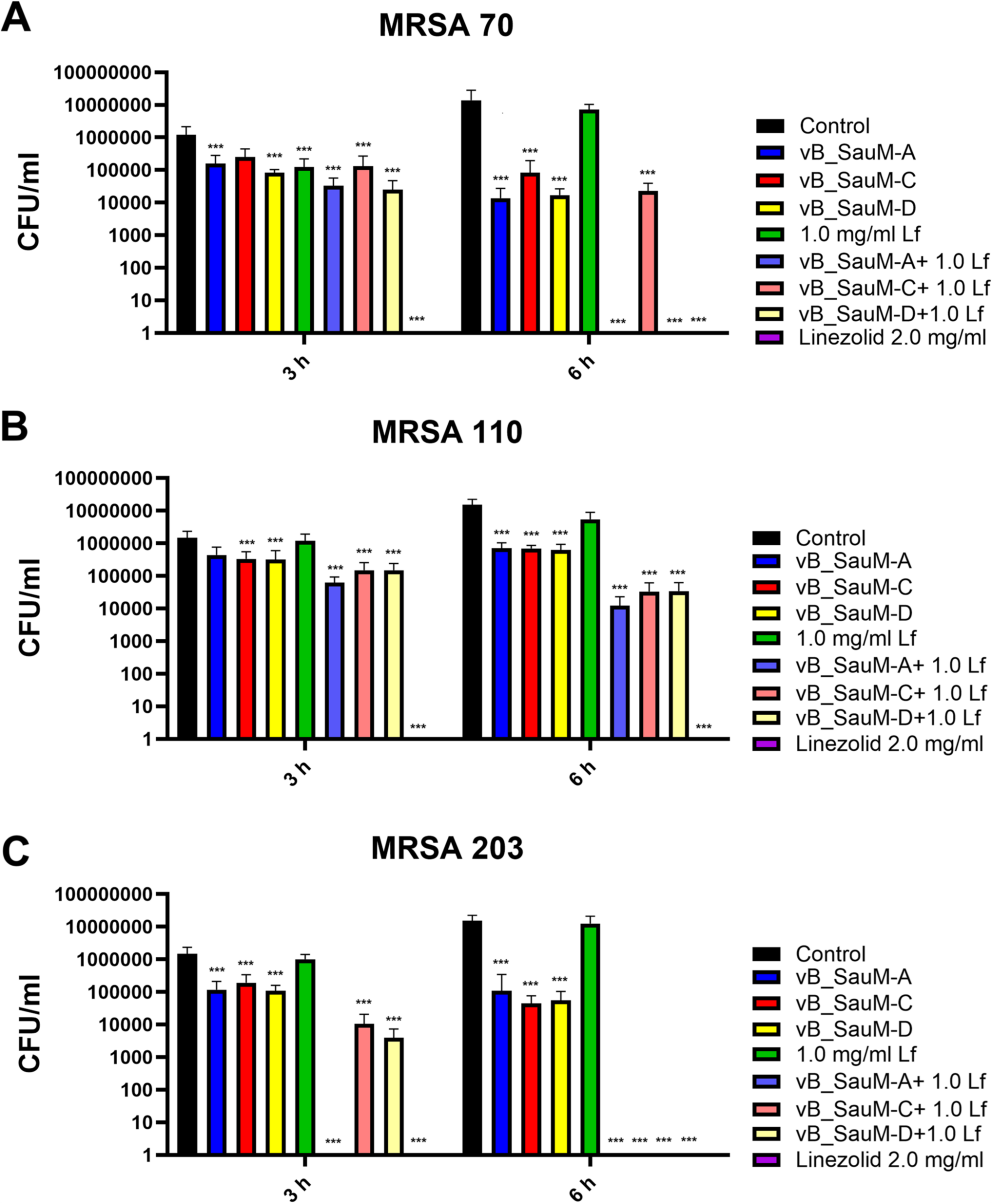
The CFU/mL of MRSA strains 70 (**A**), 110 (**B**), and 203 (**C**) in infected A549 cell line after treatment with phages vB_SauM-A, vB_SauM-C, and vB_SauM-D, lactoferrin, linezolid, or phage + Lf combination after 3 h and 6 h post-treatment. Arithmetic mean of triplicates with error bars representing SD. Statistical analysis was performed using the Kruskal–Wallis test followed by Dunn’s multiple comparison test for values with nonparametric distribution, with ***p < 0.001

In order to evaluate the efficacy of phage, lactoferrin, or combination treatment against three MRSA strains, the infected cell line model was implemented (Piechowicz et al. 2024). After the first 3 h of treatment, a significant reduction in bacterial load was observed for the majority of tested combinations, with phages combined with lactoferrin proving more effective than phages alone. The treatment with linezolid resulted in a reduction of bacterial titer below detection levels for all tested MRSA strains (Fig. 2). Six hours of treatment of MRSA 70 with vB_SauM-A + Lf and vB_SauM-D + Lf and MRSA 203 with vB_SauM-A + Lf resulted in a decrease of bacterial CFU/ml below the detection level (~ 100 CFU/mL), making these treatments as effective as linezolid (Fig. 2 A and C). Six hours of treatment with phage-lactoferrin combination of cells infected with MRSA 110 resulted in reduction of bacterial load to ~ 5 × 10^4^ CFU/ml, while phages alone reduced the load to ~ 10^6^ CFU/ml in comparison (Fig. 2B).

### Influence of phage and lactoferrin treatment of infected cell culture on cell viability

**Fig. 3:**
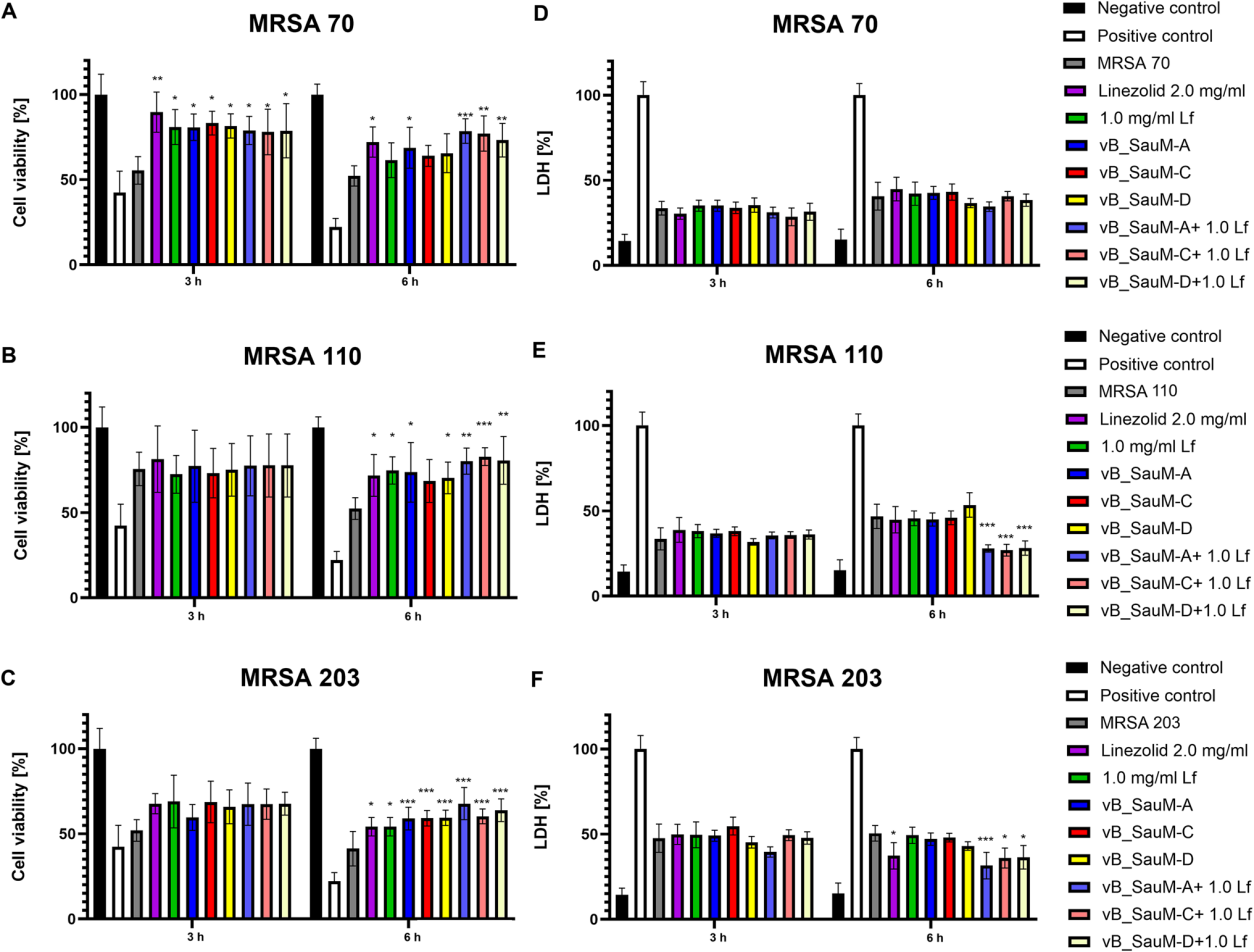
Cell viability (**A**, **B**, **C**) and LDH release (**D**, **E**, **F**) from the infected A549 cells treated with phages vB_SauM-A, vB_SauM-C, vB_SauM-D, 1.0 mg/mL lactoferrin, 2.0 mg/mL linezolid, and phage + Lf combination in comparison to the untreated negative control and positive control. Arithmetic mean of triplicates with error bars representing SD. Statistical analysis was performed using one-way ANOVA, with ***p < 0.001, **p < 0.01, and *p < 0.05

The influence of treatment on A549 cells was performed using neutral red staining, as well as LDH release assay. We have observed that in the case of MRSA strain 70 after 3 h, all used treatments were able to inhibit the toxic effect of *S. aureus* infection, observed as a decrease in cell viability. After 6 h, only linezolid, vB_SauM-A, and phage + Lf treatments were able to inhibit further viability decline (Fig. 3A). LDH level analysis revealed no significant differences between treated cultures and untreated control (Fig. 3B). For MRSA 110 infected cells, inhibition of bacterial infection on A549 cells was observed for all applied treatments, except for vb_SauM-C, after 6 h, but the statistical significance was higher in the case of phage + Lf treatment (Fig. 3C). Furthermore, after 6 h of treatment, a significant difference was also observed in LDH levels between infected control and phage + Lf-treated cells (Fig. 3D). A similar observation was made in the case of MRSA 203; significant inhibition of bacterial toxic effect on A549 cells was observed for all treatment combinations only after 6 h, with pure phage and phage + Lf treatment showing higher statistical significance than the use of linezolid or lactoferrin alone (Fig. 3E). LDH levels were significantly lower only in the case of phage-Lf-treated cells after 6 h when compared with the control (Fig. 3F).

### Influence of phage and lactoferrin treatment of infected cell culture on inflammasome activation

**Fig. 4:**
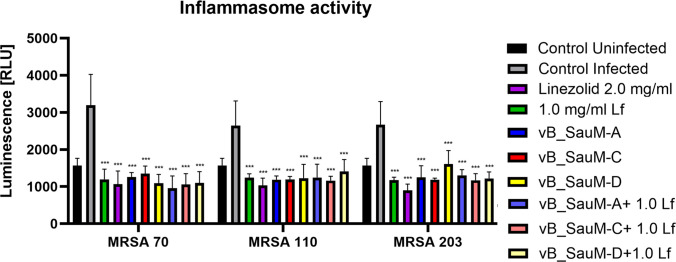
Inflammasome activation measured by caspase-1 activity in the infected A549 cells treated with phages vB_SauM-A, vB_SauM-C, vB_SauM-D, 1.0 mg/mL lactoferrin, 2.0 mg/mL linezolid, and phage + Lf combination in comparison to the untreated negative control and positive control. Arithmetic mean of triplicates with error bars representing SD. Statistical analysis was performed using the Kruskal–Wallis test followed by Dunn’s multiple comparison test for values with nonparametric distribution, with ***p < 0.001

The inflammasome activation in A549 cells measured by caspase-1 activity after 6 h of treatment. Caspase-1 was selected due to its commonly increased activity during *S. aureus* infection (Soong et al. 2012; Rasmussen et al. 2019). We observed that all treatments (linezolid, lactoferrin, phages, or phages + Lf) resulted in significantly reduced caspase-1 activity compared to the infected, untreated control. In cells infected with MRSA 70, the treatment with linezolid or phage + Lf resulted in the lowest luminescence values. For MRSA 110-infected cells, linezolid, followed by vB_SauM-C + Lf, was the most effective. Similar results were observed in MRSA 203 infection, followed by vB_SauM-D + Lf and vB_SauM-C (Fig. 4).

### Assessment of phage transcytosis through pulmonary fibroblasts

**Fig. 5:**
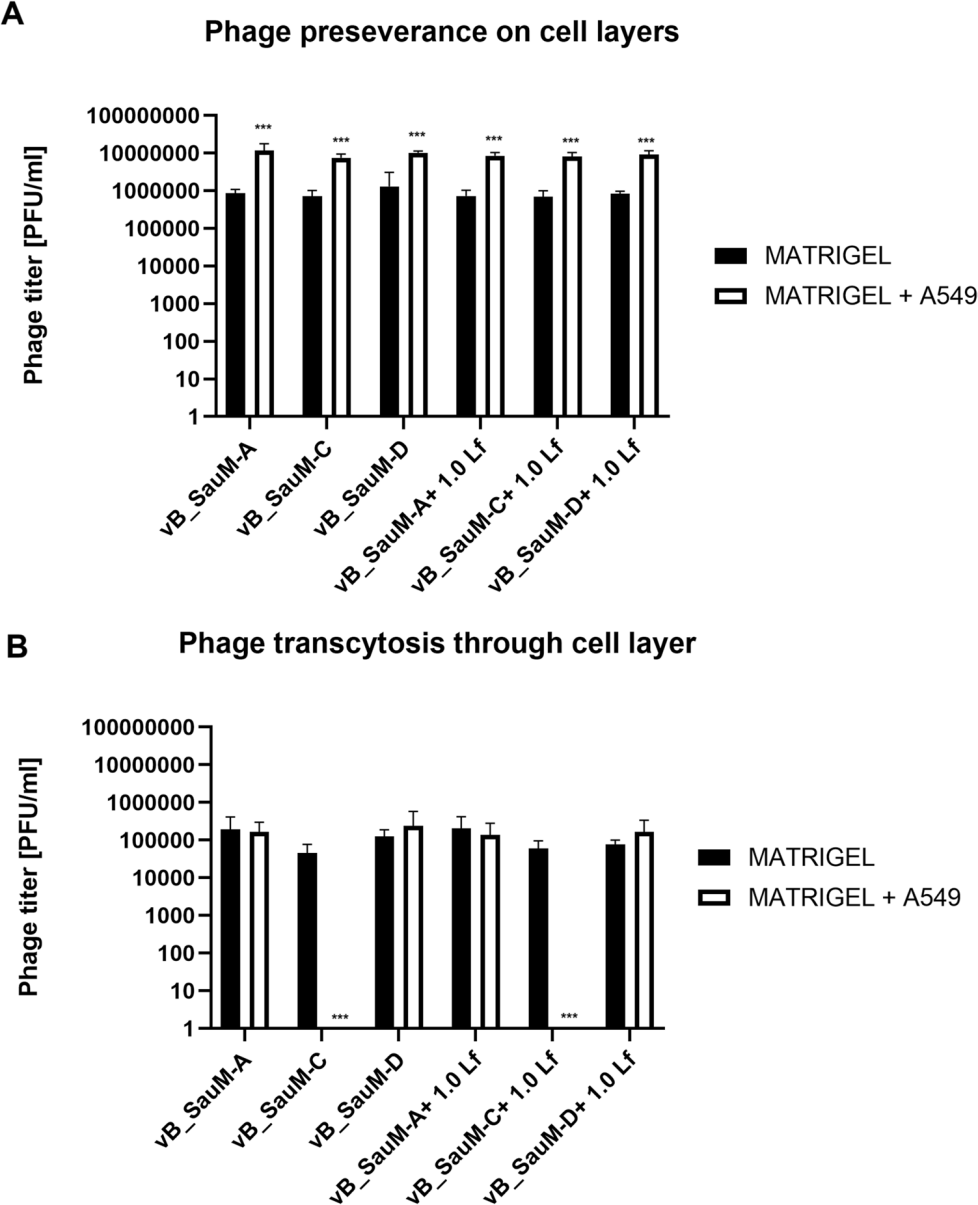
The perseverance on cell layer (**A**) and transcytosis (**B**) of phages vB_SauM-A, vB_SauM-C, vB_SauM-D alone or supplemented with 1.0 mg/mL lactoferrin. Arithmetic mean of triplicates with error bars representing SD. Statistical analysis was performed using *t*-test, with ***p < 0.001

In addition to investigating the effects of phages and lactoferrin on cell viability and inflammatory response, we examined whether three Kayviruses are capable of penetrating A549 cells and whether supplementation with lactoferrin influences this process by either enhancing or inhibiting phage penetration. We have examined whether phages (with and without Lf supplementation) are capable of penetrating A549 cells, and if after passing through the cell layer seeded on the insert, could penetrate the layer of cells seeded on the bottom of the multiwell plate (See Sect. ”Phage transcytosis”). Since Matrigel® was used in our experiments, a preliminary study aimed to assess whether the phages could traverse Matrigel® layer without losing infectivity. We observed that the phages remained active after contact with Matrigel® and were able to freely pass through it (Fig. 5 A and B). Statistically significant difference was detected in phage persistence on the cell layer in the upper chamber between pure phage lysates and phages supplemented with lactoferrin, with phage + Lf combination resulting in improved phage preservation (Fig. 5A). During analysis of phage passage through cells, phage vB_SauM-C was not recovered from the medium collected from the lower chamber, despite its ability to pass through the Matrigel® alone, and the supplementation with Lf did not cause any changes. In contrast, for phages vB_SauM-A and vB_SauM-D, there were no significant differences between the levels of phage transcytosis between Matrigel® alone or Matrigel® coated with A549 (Fig. 5B). The PFU/mL of phages passing through the cell layer ranged approximately between 1 and 2 × 10^5^, corresponding to about 0.015% of the initial phage dose used in the experiment. No phages were recovered from the cells seeded at the bottom of the multiwell plate.

## Discussion

Outbreaks of viral respiratory diseases such as influenza, SARS-CoV-1, and SARS-CoV-2 often lead to high mortality not only from the viruses themselves but also from secondary bacterial infections, primarily pneumonia and bloodstream infections, with *S. aureus* being one of the leading infective agents (Piechowicz et al. 2024; Borgogna et al. 2018). In the last 50 years, we observe a growing number of infections caused by methicillin-resistant strains which adds to their severity (Calle et al. 2016; Delaney et al. 2008; Rønning et al. 2025). Current treatment protocols typically involve the use of linezolid or vancomycin (Clark and Hicks 2024). However, although effective, these treatments are known to cause side effects, the most notable being thrombocytopenia, that can affect up to 30% of the linezolid-treated patients (Zhang et al. 2023; Qamariat et al. 2024; Thabit et al. 2024). Others include optic neuropathy, peripheral neuropathy, lactic acidosis, or bone marrow suppression (Kishor et al. 2015; Shahbazi 2025). Therefore, treatments other than antibiotics should be available for the patients with secondary bacterial infections, especially if they are in the risk group for antibiotic-related side effects development.

In our work, we combined three Kayviruses with lactoferrin and tested the efficacy and safety of the combinations in vitro, using infected pulmonary epithelial cell cultures as a model, as we have previously observed that the addition of Lf increased phage efficacy and influenced phage life cycle (Kosznik-Kwaśnicka et al. 2022; Grzenkowicz et al. 2025b). We have observed that neither the phages, lactoferrin, nor any tested combination influenced the viability of uninfected A549 cell line, in contrast to linezolid. In terms of inflammasome activation, we have observed that in an uninfected culture, we did observe a significant drop in caspase-1 activity in linezolid-treated cells. However, this decrease may be a result of a lower number of viable cells in tested samples, as linezolid caused a 20% drop in cell viability.

During the experiment with infected A549 cell culture, we observed that supplementing phages with lactoferrin enhanced their antibacterial efficacy, particularly after 6 h of treatment. In general, phages were less effective than linezolid, except for the combination of phage vB_SauM-A with Lf. However, the tested Kayviruses were less efficient in the reduction of CFU/ml of MRSA strains than they were in the case of *S. aureus* strains isolated from patients with COVID-19. Here, in 5 out of 10 cases, the phages were able to reduce the bacterial load below the level of detection after 4 h of treatment (Piechowicz et al. 2025). In MRSA-infected A549 cell culture, only phages vB_SauM-A and vB_SauM-D were able to reduce the bacterial load below detection level after 6 h of treatment and in combination with 1.0 mg/mL Lf. Additionally, phage treatment was able to counteract the negative effect of *S. aureus* on cell viability, which corresponds with other reports (Piechowicz et al. 2025; Yin et al. 2017; Shan et al. 2018).

Lactoferrin is known for its anti-inflammatory activity, while phages are reported to be able to act as both anti- or pro-inflammatory agents (Conneely 2001; Drago-Serrano et al. 2017; Górski et al. 2023). Therefore, we analyzed the effects of vB_SauM-A, vB_SauM-C, and vB_SauM-D treatment of the infected A549 cells on caspase-1 activity and whether combining the treatment with lactoferrin would result in any changes. We have observed that tested phages were able to reduce the inflammatory response, similarly to other staphylococcal phages (Zhang et al. 2018; Suda et al. 2022). The addition of lactoferrin to phage lysate did not cause any further decrease in caspase-1 activity. However, as the phages alone were able to reduce the inflammasome activity below the level of uninfected control, it is possible that obtaining even lower values was simply impossible. To assess this, other phage-lactoferrin combinations should be tested in the future, and further studies should also consider other cytokines to provide deeper insight into the potential modulation of inflammatory response by phage-Lf treatment.

Finally, the phage interactions with eukaryotic cells, especially phage ability to pass through cell layers, cause concerns of phage therapy safety, as the phages could possibly spread throughout the organism of a patient in an uncontrolled manner (Nguyen et al. 2017; Bichet et al. 2021). Although bacteriophages are unable to replicate in the absence of their bacterial hosts within the patient, there is concern that their translocation across cellular barriers and subsequent dissemination may trigger an immune response (Bollyky and Secor 2019; Varadan and Grasis 2024). It has been observed that phage penetration through cells seems to depend on a variety of factors, such as the type of phage used, the type of eukaryotic cells, and, in terms of in vivo studies, the way phages are introduced into the system (Bichet et al. 2021; Dąbrowska and Abedon 2019). We have observed that two out of three tested phages, vB_SauM-A and vB_SauM-D, were able to pass through the A549 cell layer. Phage vB_SauM-C was able to pass through Matrigel® alone but was not recovered when the matrix was coated with cells, suggesting it could pass through the extracellular matrix, but not the cells themselves. However, none of the phages was detected in the basal layer of A549 cells suggesting limited spread through the system. The supplementation with Lf did not result in an increase or decrease in phage survival on the cell layer or transcytosis. Therefore, it could be assumed that using phages in combination with lactoferrin in the respiratory tract should not result in increased phage distribution through the system.

Considering both our findings (current and prior) and the available literature, it can be assumed that the incorporation of Lf into phage lysates or cocktails could have a beneficial effect on therapeutic outcome (Zimecki et al. 2008; Kosznik-Kwaśnicka et al. 2022; Grzenkowicz et al. 2025a). However, although the available data are promising, they remain limited, and additional studies are required to confirm this hypothesis. Future investigations should include a broader range of phage types and host species, as well as different types of eukaryotic cells. Furthermore, the phage + lactoferrin influence on inflammatory response should be tested on animal models in order to fully understand the possible effect of this combination on the immune response of the patient.

## Data Availability

Raw data are available at Polish Platform of Medical Research 10.60816/5hmy-5q52.
